# Development of Real Time PCR to Study Experimental Mixed Infections of *T. congolense* Savannah and *T. b. brucei* in *Glossina morsitans morsitans*

**DOI:** 10.1371/journal.pone.0117147

**Published:** 2015-03-04

**Authors:** Heba A. Ahmed, Ewan T. MacLeod, Susan C. Welburn, Kim Picozzi

**Affiliations:** 1 Division of Pathway Medicine, School of Biomedical Sciences, College of Medicine and Veterinary Medicine, University of Edinburgh, Edinburgh, United Kingdom; 2 Faculty of Veterinary Medicine, Zagazig University, Zagazig, Ash Sharqiyah, Egypt; University of Cincinnati, UNITED STATES

## Abstract

Tsetse flies are able to acquire mixed infections naturally or experimentally either simultaneously or sequentially. Traditionally, natural infection rates in tsetse flies are estimated by microscopic examination of different parts of the fly after dissection, together with the isolation of the parasite *in vivo*. However, until the advent of molecular techniques it was difficult to speciate trypanosomes infections and to quantify trypanosome numbers within tsetse flies. Although more expensive, qPCR allows the quantification of DNA and is less time consuming due to real time visualization and validation of the results. The current study evaluated the application of qPCR to quantify the infection load of tsetse flies with *T. b. brucei* and *T. congolense* savannah and to study the possibility of competition between the two species. The results revealed that the two qPCR reactions are of acceptable efficiency (99.1% and 95.6%, respectively), sensitivity and specificity and can be used for quantification of infection load with trypanosomes in experimentally infected *Glossina morsitans morsitans*. The mixed infection of laboratory *Glossina* species and quantification of the infection suggests the possibility that a form of competition exists between the isolates of *T. b. brucei* and *T. congolense* savannah that we used when they co-exist in the fly midgut.

## Introduction

Tsetse flies are responsible for the transmission throughout sub-Saharan Africa of African trypanosomes, causative agents of sleeping sickness (human African trypanosomiasis) and nagana (animal African trypanosomiasis). Establishment within the tsetse can occur when the fly takes a bloodmeal from an infected animal. However, not all infected bloodmeals will lead to establishment of a trypanosome population in the tsetse. Several factors have been shown to be important in the susceptibility of the fly to infection. These include temperature [1], age of the fly at the time of exposure [2], host factors [3], fly/trypanosome combinations [4], symbiont presence [5], antimicrobial peptides [6], and more recently the effect of oxidative state in the midgut [7] and cyclic nucleotides [8].

The majority of experiments conducted so far on the establishment of trypanosomes within tsetse flies usually score the infection as either positive or negative; they do not take into account how heavy or light the residential trypanosome population is. There have, however, been several papers where the population of trypanosomes has been quantified [9]. The technique which has been used most widely is haemocytometer counts of midgut homogenates. While a more recent laboratory-based study quantified populations using fluorescently labeled trypanosomes [9], this approach, however, is unsuitable for the study of wild tsetse flies with residential trypanosome infections.

To date there have been few investigations into the dynamics of a trypanosome infection during the initial stages of infection (*i*.*e*. how does the population grow once it enters the fly). The study by Peacock *et al*. [9] showed that upon imbibement of an infected bloodmeal, there is gradual growth in numbers of the two strains of *Trypanosoma brucei brucei* that were investigated until day six when two outcomes become apparent, either the tsetse will develop a viable infection or the fly will clear the protozoa. This causes problems identifying a fly as being infective as it is difficult to distinguish between an establishing or dying population within the first week of exposure. Further problems may also occur with PCR as this technique will identify genomic fragments, which may be retained within the gut several days after the last live trypanosome has perished.

A number of studies have investigated the occurrence of mixed infections in experimentally infected tsetse using PCR to confirm trypanosome infection status [10], [11], [12], [13], [14]. However, none of these studies has quantified the numbers of trypanosomes present within the tsetse flies in these mixed infections.

Here we report the development of qPCR reactions that can be used to enumerate the populations of insect form trypanosomes in culture and in tsetse flies. Two different qPCR reactions were developed, for *Trypanosoma brucei brucei* s.l. the single copy gene glycosyl-phosphatidylinositol-specific phospholipase C (GPI-PLC) qPCR [15] was used, while for *T. congolense* a mini-chromosome target was used [16]. Once optimised these reactions were used in comparison to haemocytometer counts to estimate the number of trypanosomes in midgut homogenates of laboratory infected tsetse flies. Moreover, the protocols have been applied to study mixed infections of *T. brucei* and *T. congolense* obtained from both simultaneous and sequential infections. The significant correlation between the two methods suggests that either haemocytometer or qPCR can be used for the quantification of trypanosomes and that both are comparable, permitting the application of these protocols to determine trypanosome load within flies with single infections in both high and low resource settings.

## Materials and Methods

### Tsetse fly and trypanosome isolates

*Glossina morsitans morsitans* (Westwood) were originally from the Langford colony established at Bristol from pupae collected from Zimbabwe in 1967; the colony had been at University of Edinburgh for 10 years when the experiments were carried out. Tsetse flies were kept at 25°C ± 1°C and at 55–60% relative humidity.

Two trypanosome isolates were used in the current work, namely *T. b. brucei*, Buteba 135 and *T. congolense* savannah, Sikuda 124. Both of these isolates were collected in Uganda from cattle during 1990. Material derived from procyclic cultures was used to optimise the qPCR reactions. This material was obtained by infecting flies with bloodstream form trypanosomes, dissection of flies followed by culture of infected midguts in Cunningham’s medium as described by Maudlin [17].

For control purposes DNA from a previously identified positive infection of *T. vivax* was included during the development of the qPCR methodology. DNA from procyclic forms and from midguts was extracted by DNeasy Blood and Tissue Kits (Qiagen).

### *T. brucei* s.l. qPCR using a single copy gene target

Primer sets for the amplification of the single copy PLC gene specific for *T. brucei* s.l were used [18]. The amplification mixture contained 1 μl DNA template, 200 nM of each primer (PLC1: 5’- CGC TTT GTT GAG GAG CTG CAA GCA-3’ and PLC2: 5’- TGC CAC CGC AAA GTC GTT ATT TCG-3’) [18], 5 μL SYBR Green I ready-made master mix supplied by Qiagen supplemented with 0.1 units AmpErase [Uracil N-glycosylase] (Applied Biosystems).

The reaction conditions used were 50°C for two minutes to activate the AmpErase, 95°C for 15 min then 40 cycles at 94°C for 30 sec, 63°C for 90 sec and 72°C for 70 sec followed by plate read for fluorescence acquisition. A temperature gradient between 55°C and 95°C was run to obtain the dissociation curve. The standards and samples were run in triplicates and expressed as a mean value; non-template controls were also used to check the presence of contamination.

The sensitivity of the assay was evaluated using different amounts of *T. b. brucei* DNA by serial dilution in Cunningham’s media. The starting concentration was confirmed by direct visualisation, in a haemocytometer, as four parasites per millilitre. The serial dilutions were prepared and validated through 10 orders of magnitude prior to DNA isolation, the standard curve is shown in S1 Fig. The amplification efficiency was not compromised when materials containing a dual infection, at a background concentration of 5 ng per ul, were assessed the presence of a single parasitic target.

Melting curve analysis was performed, no multiple peaks were observed; to confirm the specificity of the reactions in mixed infection, an agarose gel was run to further ensure that only the target was amplified.

### Optimisation of *T. congolense* savannah qPCR

Primer sets that had been previously used for the amplification of *T. congolense* savannah [16] were chosen for the qPCR reaction in the current study. Each PCR reaction contained 1 μl of the extracted DNA, 300 nM of each primer (TCS-RF: 5’-GGA CAA ACA AAT CCC GCA CA-3’ and TCS-RR: GAG AAC GGG CAC TTT GCG A-3’) and 5 μl iQ^TM^SYBR Green I supermix (Bio-Rad). Water was added to a final volume of 10 μl.

The cycling programme was initiated with denaturation of DNA and polymerase activation at 95°C for 10 min followed by 30 cycles each of 94°C for 20 sec and 60°C for one minute followed by plate read for fluorescence acquisition. The sensitivity of the assay was evaluated using different amounts of *T. congolense* savannah DNA by serial dilution following the protocol described above. The standards and samples were run in triplicates and expressed as a mean value, non-template controls were also used to check the absence of contamination, the standard curve is shown in S2 Fig. As above, the amplification efficiency was not compromised in the presence of a dual infection at a background concentration of 5ng per ul; multiple peaks were confirmed as being absent from the reaction through melting curve analysis and visualization of the amplification product.

### Normalisation of the qPCR reactions using *Glossina* DNA as an internal control

An internal control was introduced to each of the purified trypanosome standards; this ensured that variation in the amplification profile observed across the dilution series was a true representation of the target material present. This was achieved by the addition of a known quantity of tsetse DNA, such that its quantification could be taken as an amplification constant within the standard curve.

The NanoDrop quantification of the *Glossina* DNA extracted from non-infected flies revealed an average of 2600 ng/fly (13 ng/μl). Primers targeting the alpha-elongation factor specific for *Glossina* species were used for qPCR. The sequences of the primers are TseAF: 5'-CGG CTG GCA CGG TGA TAA CAT-3' and TseAR: 5'-GCG GGA GGG TGG CAA CATT-3', they amplify 121 bp amplicon.

The amplification mixture contained 13ng DNA template, 300 nM of each primer, 5 μL SYBR Green I ready-made master mix supplied by Qiagen (containing HotStarTaq DNA Polymerase, Quantitect SYBR Green I PCR Buffer [Tris-Cl, KCl, (NH4)2SO4, 2.5 mM MgCl2, pH 8.7], 0.2 mM dNTP mix,SYBR Green I dye, ROX dye), 0.1 units AmpErase [Uracil N-glycosylase] (Applied Biosystems). Water was added to a final volume of 10 μl.

The reaction conditions used were 50°C for two minutes to activate UNG, 95°C for 15 min then 40 cycles at 95°C for 45 sec, 57°C for 45 sec and 72°C for 45 sec, plate read for fluorescence acquisition was set up after the extension step following the first 15 cycles to get rid of fluorescence noise that occurs before the amplification of the product. A temperature gradient between 55°C and 95°C was run to obtain the dissociation curve. The standards and samples were run in triplicates and expressed as a mean value, non-template controls were also used to check the presence of contamination, the standard curve is shown in S3 Fig.

An average Ct value of 28.7± 0.7 was obtained for this internal control, from all serial dilutions of *T. b. brucei* and *T. congolense* savannah that had spiked with the constant amount of *Glossina* DNA. The calculated average Ct value was equivalent to 13.2 ng/μl of *Glossina* DNA.

### Infection of tsetse flies with procyclic forms

Teneral male tsetse flies (less than 24 hours old) in groups of 30 had their wings clipped and were allowed to rest for 24 h before being infected with trypanosomes. Cultured procyclic forms were centrifuged at 2500 rpm for 5 min and the culture supernatant was removed, trypanosomes were then resuspended in 5 ml of ovine blood at a concentration 1.67x10^6^ /ml which is equivalent to 50,000 parasites per blood meal.

The infection rate within this tsetse colony was <20% [7]. For practical reasons, steps were taken to increase the midgut infection rate to 100%; for this purpose infective blood meals were supplemented with 15 mM GSH [7]. The infective bloodmeal was placed on a heated tray and covered with a silicon membrane. Tsetse were then introduced onto the membrane and allowed to feed. Flies that did not feed were removed from the experiment.

Five groups of *G. m. morsitans* in groups of 30 were infected to evaluate the suitability of qPCR in quantifying trypanosome infections of the flies when infected with single and mixed species.

The first group of flies were infected with a single infection of *T. b. brucei*; the second group of flies were infected with a single infection of *T. congolense* savannah, the third group of flies were infected simultaneously with *T. b. brucei*/*T. congolense* savannah; the fourth group of flies were infected firstly with *T. b. brucei* and after two days another feed infected with *T. congolense* savannah; finally, the fifth group of flies were infected firstly with *T. congolense* savannah followed by *T. b. brucei* after two days.

### Dissection of the flies and collection of material

Tsetse flies were chilled at 4°C for 30 min and washed in 5% sodium hypochlorite followed by sterile water and then kept on ice until dissection. Flies were dissected in saline 15 days post-infection, midguts were then placed in a micro-centrifuge tube containing 200 μl PBS and homogenised using disposable micro-tube pestles. At 15 days post infection trypanosome infections resulting from the infection bloodmeal would have established within the midgut, with sufficient time having elapsed to ensure the degradation of DNA from any non-viable parasites. Twenty microliters retained for counting trypanosomes using a haemocytometer while the remaining 180 μl was used for DNA extraction. DNA was extracted from samples using a DNeasy Blood and Tissue kits (Qiagen).

### Statistical analysis

The number of parasites present in midgut homogenates was calculated using the method described in Welburn and Maudlin in 1997 [19].

The qPCR amplification efficiency (E) determined by linear regression of the standard curve was calculated from the slope (s) of the standard curve using the equation: E = 10-1/s-1 [20]. The acceptable efficiency of the qPCR assay should be between 90–110%. Inter assay precision was calculated using the following formula: Inter-assay precision = (Standard deviation of the mean Ct of the triplicates/ Grand mean Ct of the triplicates) x 100 [21]. Normality of the data distribution was tested using the *Anderson-Darling* normality test in Minitab version 15 (Minitab, Inc.). The data were considered not normally distributed when p value was <0.05.

The number of the parasites was calculated by plotting the average Ct values versus the log (10) of the parasite concentration/ml. Correlation between haemocytometer and qPCR quantification and *Mann-Whitney U* test were calculated using Minitab version 15 (Minitab, Inc.).

*Kruskal-Wallis* one-way analysis of variance (ANOVA) was used to compare between the median of the observed groups, and *Dunn’s* multiple comparison test was used to test the difference in between the four groups as a multiple comparison post-test. *Kruskal-Wallis* and *Dunn’s* multiple comparison tests were calculated using GraphPad Prism, Inc., version 5.02.

## Results

The current work aimed to evaluate and investigate the use of qPCR in the determination of midgut populations of *T. b. brucei* and *T. congolense* savannah in single and mixed infections and studying the relationship between the two parasites in the fly midgut.

Trypanosome DNA standards were spiked with a constant amount of *Glossina* DNA to evaluate if the fluorescence detected using *T. b. brucei* and *T. congolense* savannah qPCR reactions was a result of the amplification of trypanosome DNA and to ensure that any signal recorded was not due to the presence of *Glossina* DNA. The alpha-elongation factor specific for the host *Glossina* species was used as an internal control throughout (this data is not shown).

### *T. brucei* s.l. qPCR using a single copy gene target

The efficiency of GPI-PLC as a qPCR target was calculated from the slope of the line generated from the average standard curve of cycle threshold (Ct) versus log10 of the parasite concentration per ml, the result was 99.1%. A high Pearson correlation coefficient (R^2^ = 0.9996) was obtained indicating a linear standard curve; this implies that the efficiency of amplification was consistent at varying template concentrations.

The inter assay precision was calculated in 18 repeats of standards and found to be less than 10% (4.2–7.6%) which is within the acceptable range. This indicates the reproducibility of the assay over six orders of magnitude from 4x10^6^ to a concentration of 40 parasites per ml; showing minimal variation and high precision.

### Optimisation of *T. congolense* savannah qPCR

Primers designed to target a satellite DNA sequence of 5400 copies within *T. congolense* savannah were used in the current study; amplification of this target consistently generated a single band of 316 bp. A high Pearson correlation coefficient (R^2^ = 0.9959) indicates a linear standard curve; the efficiency was calculated as described above, and was found to be 95.6%.

The inter assay precision was calculated in 20 repeats of standards and found to be less than 10% (2.2–6.9%). This indicates the reproducibility of the assay over six orders of magnitude from 4x10^6^ to 40 parasites per ml; with the minimal variation and high precision of the applied assay.

### Reaction specificity

The specificity of these reactions was determined using different concentrations of other trypanosome DNA including where appropriate *T. brucei, T. congolense* savannah and *T. vivax*, and using also different concentrations of *G. m. morsitans* DNA. Combinations of infectious agent were also assessed, to review the influence of concurrent infections on the efficiency of the species specific reaction.

The results showed that each reaction was species specific and that the presence of additional genetic material has very little influence on the amplification profile of the reaction (see also S1 and S2 Figs.). While the profile for *T. brucei* is unaffected across the concentration gradient, slight variation is seen within the *T. congolense* savannah profile at the lower levels of parasitaemia. It is noted that these differences are within no more than 0.9 SD of the average crossing threshold.

### Comparison of qPCR and haemocytometer quantification

Flies which were infected with either *T. b. brucei* or *T. congolense* savannah were dissected 15 days post-infection and the infection load estimated using both haemocytometer and qPCR quantification.

**Fig 1:**
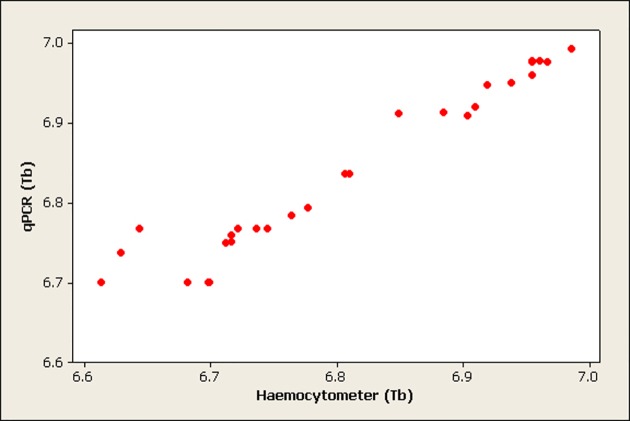
Scatterplot showing the correlation between qPCR and haemocytometer quantification of *T. b. brucei* (Tb) in midguts (numbers are presented on log 10 scale and each dot is the average of three replicates).

**Fig 2:**
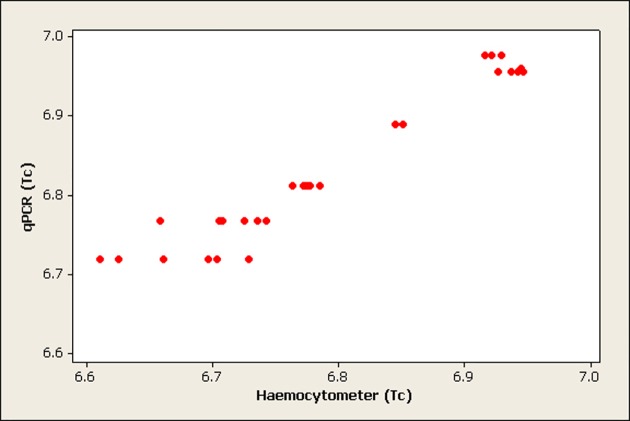
Scatterplot showing the correlation between qPCR and haemocytometer quantification of *T. congolense* savannah (Tc) in midguts (numbers are presented on log 10 scale and each dot is the average of three replicates).

The correlation between haemocytometer and qPCR quantification was calculated and the results revealed a significant correlation (p<0.001) between the two methods in quantifying *T. b. brucei* (Pearson correlation = 0.969) and *T. congolense* savannah (Pearson correlation = 0.968). Figs. 1 and 2 show scatter plots of the correlation between qPCR and haemocytometer quantification of *T. b. brucei* and *T. congolense* savannah respectively.

**Fig 3:**
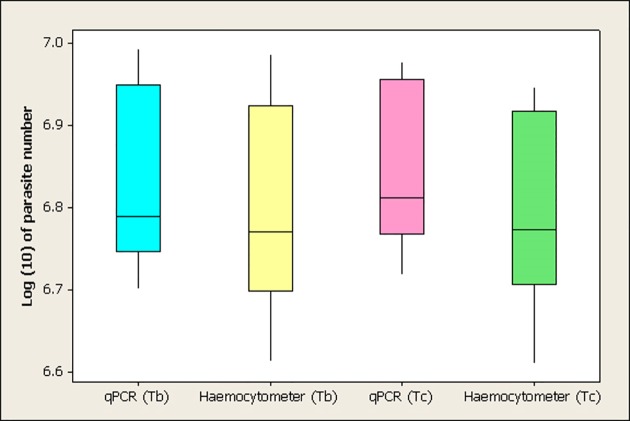
Boxplot showing the median log (10) number of parasites (Tb: *T. b. brucei*, Tc: *T. congolense* savannah) quantified using qPCR and haemocytometer in the single infected groups (Tb by qPCR: 6.79, Tb by Haemocytometer: 6.77, Tc by qPCR: 6.81, Tc by Haemocytometer: 6.77). Numbers are presented on the log 10 scale, each boxplot shows the median value and the upper and lower quartiles.

The detection limit of qPCR and haemocytometer to *T. b. brucei* and *T. congolense* savannah was compared using Mann-Whitney U test. Fig. 3 shows a boxplot of the log (10) number of parasites/midgut quantified using qPCR and haemocytometer for the two trypanosome species. The median population of *T. b. brucei* was quantified with qPCR as 6.79 (6.2x10^6^), which is insignificantly higher than the median log number of parasites (6.77 = 5.9x10^6^) obtained using haemocytometer method (p = 0.18). Moreover, the median number of *T. congolense* savannah obtained by qPCR (6.81 = 6.5x10^6^) was not significantly higher than the median number (6.77 = 5.9x10^6^) obtained with haemocytometer quantification (p = 0.1).

### Infection load of *Glossina* with *T. b. brucei* single and mixed infections

The infection load of *G. m. morsitans* with *T. b. brucei* when in single and mixed infection was compared in the four fly groups; as detailed as groups 1, 3–5 within the methodology section.

S4 Fig. shows a boxplot of the *T. b. brucei* quantification in the four groups. The median number of *T. b. brucei* in the single infection group was 6.79 (6.2x10^6^), while it was 6.51 (3.3x10^6^), 6.77 (5.9x10^6^), and 1.75 (6.3x10) in simultaneous *T. b. brucei*/*T. congolense* savannah, sequential *T. b. brucei*/*T. congolense* savannah and sequential *T. congolense* savannah/*T. b. brucei* infection group, respectively. The infection rate in the four groups was 100% for both species. There was a significant difference in the median of *T. b. brucei* number in the four groups (H_3_ = 74.03, p<0.001). There was no statistical significant difference between the median number of *T. b. brucei* when present in single infection, when fed simultaneously and when first fed in the sequential infection (p>0.05). However, the median number of *T. b. brucei* fed secondly in the sequential infection with *T. congolense* savannah was significantly lower than the other three groups (p<0.05).

### Infection load of *Glossina* with *T. congolense* savannah single and mixed infections

The infection load of *G. m. morsitans* with *T. congolense* savannah was compared in the four groups, as detailed as groups 2–5 within the methodology section.

It was observed that none of the flies infected sequentially with *T. congolense* savannah following exposure to *T. b. brucei* 48 hours earlier had detectable *T. congolense* savannah infections. S5 Fig. shows a boxplot for the *T. congolense* savannah quantification in the four groups.

The median number of *T. congolense* savannah in the single infection group was 6.81 (6.5x10^6^); while it was 4.1 (1.14x10^4^) and 6.81 (6.5x10^6^) in the simultaneous *T. b. brucei*/*T. congolense* savannah and sequential *T. congolense* savannah/*T. b. brucei* infection group, respectively. The infection rate in the single, simultaneous and sequential *T. congolense* savannah/*T. b. brucei* was 100% for both species, while, none of the flies infected with sequential *T. b. brucei*/*T. congolense* savannah were infected with *T. congolense* savannah.

The result in S5 Fig. shows that there was a significant difference in the median of *T. congolense* savannah number in the four groups (H_3_ = 102.6, p<0.001). There was no statistical significant difference between the median number of *T. congolense* savannah when present in single infection and when first fed in the sequential infection (p>0.05). However, the median number of *T. congolense* savannah single infection was significantly higher than when fed simultaneously with *T. b. brucei* (p<0.05). Interestingly, none of the flies fed sequentially with *T. congolense* savannah in the second feed were found infected with *T. congolense* savannah.

## Discussion

The current work aimed to evaluate and investigate the use of qPCR in the determination of midgut populations of *T. b. brucei* and *T. congolense* savannah in single and mixed infections and studying the relationship between the two parasites in the tsetse midgut. Previously, Becker *et al*. [20] had developed primers targeting the satellite sequences of *T. brucei*; however, this approach was optimized for bloodstream forms diluted in blood while the current study used extracted DNA from procyclic cultures. This method was therefore not appropriate for this experimental system.

GPI-PLC was selected as for development a single copy gene specific for *T. brucei* s.l. [18]. This target was used in the current study for a qPCR reaction to quantify *T. b. brucei* in standards and *G. m. morsitans* midguts. The efficiency of the reaction was calculated to be 99.1%. The inter assay precision also showed a minimal variation and high precision of the applied assay which indicated the reproducibility of the assay over the six orders of magnitude tested. The sensitivity of the reaction was 40 parasites/ml and the reaction was specific for *T. b. brucei* with no production of secondary structures.

In the current study, primers specific for *T. congolense* savannah targeting a satellite DNA sequence [16] were modified for qPCR; the reaction efficiency was 95.6%. The inter assay precision showed minimal variation indicating the reproducibility of the assay over six orders of magnitude. The reaction sensitivity was 40 parasites/ml and the reaction was specific for *T. congolense* savannah with no tendency of forming secondary structures. There was no cross reaction of the primers with *T. brucei* s.l. and *T. vivax* (even though similar sequences of satellite DNA exist (44% and 37%, respectively for *T. brucei* s.l. and *T. vivax* when compared with *T. congolense* [22]) or *G. m. morsitans* DNA.

These techniques were applied to *in vivo* populations of trypanosomes within the tsetse midgut. Quantification of *T. b. brucei* and *T. congolense* savannah in the midguts of *G. m. morsitans* with single infections were estimated using haemocytometer and qPCR. The results showed that the median number of *T. b. brucei* and *T. congolense* savannah quantified with qPCR was comparable to the median number obtained using haemocytometer counts. Moreover, the significant correlation between the two methods suggested that either haemocytometer or qPCR can be used for the quantification of trypanosomes and that both methods were comparable. Although the infection levels obtained in the current work are 10 fold higher than that obtained by Welburn and Maudlin [19] and Van den Abbeele *et al*. [23] this could be accounted by the addition of glutathione to the bloodmeal, given that it increases infection rates in flies from 20% to 100% [7], it may also affect the dynamics of the trypanosome population.

Bottlenecks in trypanosome populations occur during transmission through the tsetse [24]. From our own observations of dissecting flies that have been infected with trypanosomes in a bloodmeal supplemented with GSH, the majority of flies appear to have high populations of trypanosomes. Future work could look at the trypanosome populations between flies that have been infected normally or with GSH and investigate if GSH has an impact on trypanosome populations especially during the early stages of infection. Although using the fluorescent techniques of Peacock et al. [9], may be more appropriate for this as qPCR will not be able to discriminate between alive and dead trypanosomes.

The next stage of the experiment was to examine mixed infections of *T. b. brucei* (Buteba 135) and *T. congolense* savannah (Sikuda 124) within tsetse. However, there are a number of limitations of this work which will need to be addressed in the future, for instance this research is limited to one isolate of *T. b. brucei* and one isolate of *T. congolense* savannah, therefore any differences might be isolate rather than species specific. We recommend that further isolates be examined to explore if these observations are common to *T. b. brucei* and *T. congolense* or are isolate specific; it may be for example that Sikuda 124 cannot easily establish in non teneral flies.

Experimental infection of different *Glossina* species with single and mixed trypanosome species has been investigated by a number of authors in order to understand the relation between the different trypanosomes, to understand the cycle of transmission inside the vector and to estimate the burden of tsetse flies in the epidemiology of trypanosomiasis. However, none of the reported studies quantified the number of parasites in the fly midgut.

**Fig 4:**
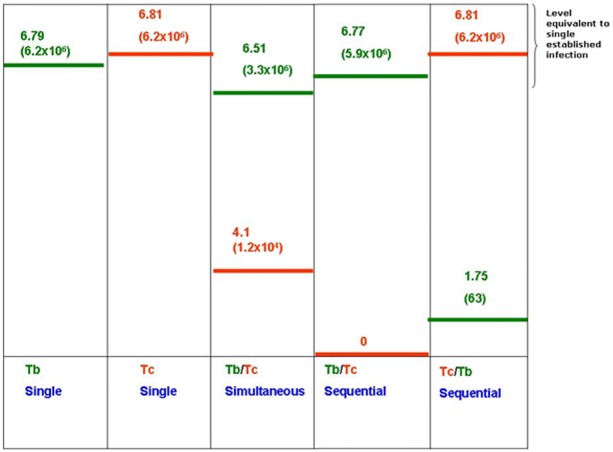
Illustration of the median log number (actual number between brackets) of *T. b. brucei* (green) and *T. congolense* savannah (red) in single and mixed infection groups of *G. m. morsitans* (Tc: *T. congolense* savannah, Tb: *T. b. brucei*).

Fig. 4 illustrates the median log number of established mid-gut communities 15 days post infection. When *T. b. brucei* or *T. congolense* savannah were fed to the flies once or in the first meal for the sequential mixed infection, the log number of parasites was consistently around 6.77 and 6.81. This confirms the ability of the parasite to establish infection in the fly midgut when present in the first meal. An observation made in agreement with expectations from previous work that reported insects taking their first bloodmeal were much more easily infected by trypanosomes [2].

However, in mixed infection groups, the log number of *T. b. brucei* was significantly higher than *T. congolense* savannah when fed to the flies in the same blood meal, this was also observed in all flies with a mixed infection regardless of which species was presented in the initially ingested infective bloodmeal. When *T. b. brucei* was fed to the flies in the first meal and *T. congolense* savannah in the second sequential meal, there were no *T. congolense* savannah infections detected at all. This suggests that the presence of *T. b. brucei* may compromise the ability of this *T. congolense* savannah to establish within the tsetse midgut.

The development of *T. b. brucei* in *G. m. morsitans* midgut did not seem to be affected by the presence of *T. congolense* in the studies of Van den Bossche *et al*. [25], [26]. This is in agreement with the current study where *T. b. brucei* developed in flies infected with *T. congolense* savannah in the first meal although the median number of *T. b. brucei* was significantly lower than the median log number of *T. congolense* savannah (p<0.05). In contrast, Kubi *et al*. [11] suggested that previous exposure of tsetse flies to an infected blood meal or the presence of trypanosome has no effect on a subsequent secondary infection.

The inability of this *T. congolense* isolate savannah to establish an infection when fed after *T. b. brucei* could be due to several reasons. It is thought that trypanosomes control their numbers in the tsetse midgut through quorum sensing [19], [27]. This could be achieved by the secretion of a factor which regulates trypanosome numbers. *T. b. brucei* may be secreting an, as yet undetected, molecule that will have a negative effect on the establishment of *T. congolense* savannah. As *T. b. brucei* has several days head start under these conditions, its numbers may have reached a quantity sufficient to prevent the establishment of *T. congolense* savannah. However, when the two species were fed simultaneously it may be that not enough *T. b. brucei* are present to affect the *T. congolense* savannah parasites which still manage to establish, albeit several orders of magnitude smaller than when *T. congolense* savannah was fed singularly. Following on from this, as *T. b. brucei* can establish a small population when fed after *T. congolense* savannah, this species may be less prone to inhibition by the presence of other trypanosome species or simply better at establishing an infection. This is also suggested by the results of the simultaneous infection where *T. b. brucei* managed to establish at higher numbers than *T. congolense* savannah.

An alternative hypothesis is the influence of a fly derived factor that is responsible for the knock-down of trypanosome numbers, with *T. congolense* savannah being more susceptible than *T. b. brucei*.

Lectins [28] and tsetse EP protein [29] have been shown to play a role in blocking establishment of trypanosome infections in the fly. As we used glutathione to reduce oxidative stress in these experiments it may be that these other molecules had an impact on *T. congolense* in non teneral flies.

Gibson and Ferris [10] designed an experiment to infect *G. m. morsitans* with simultaneous and sequential infection using *T. b. rhodesiense* and *T. congolense* savannah. The flies were dissected 20 days post-infection and examined by DNA hybridisation probes specific for the two species. The results showed that 33.3% of the flies infected simultaneously with the two species had mixed infections. However, flies with sequential infection with *T. b. rhodesiense*/*T. congolense* savannah, 54.3% of the flies had single infection with *T. b. rhodesiense* and 1.1% of the flies had single *T. congolense* savannah and mixed infection of the two species, each. In case of sequential *T. congolense* savannah/*T. b. rhodesiense*, 34.4% of the flies acquired single *T. congolense* savannah infections, while 1.04% had single *T. b. rhodesiense* and mixed infection of the two species, each. The authors concluded that it is possible to super-infect laboratory tsetse flies with *T. b. rhodesiense* and *T. congolense* savannah by sequential feeding to mimic field conditions where flies have unrestricted freedom to feed on infected hosts suggesting that mixed infections can be acquired outside the narrow window of the first feed.

In the current work, GSH was used to boost the number of midgut infections, which explains the increased number of flies with midgut infections compared to the work of Gibson and Ferris [10]. This also explains why, in the current work, 100% of flies infected with *T. congolense* savannah followed by *T. b. brucei* had infections of both trypanosome species, although the advantage of being fed first is clearly shown by the reduction in the population of *T. b. brucei* within the fly midgut. However, similar effects were evident when *T. b. brucei* was fed first followed by *T. congolense* savannah with the flies showing only *T. b. brucei* infections in the current work; by contrast only 1% of flies picked up a *T. congolense* savannah infection in the work of Gibson and Ferris [10].

The hypothesis of competition between trypanosome species was previously investigated by Seed [30] who reported evidence of competition between two clones of *T. b. gambiense* when inoculated simultaneously into rats. One clone outgrew the other to an extent disproportionate to the difference in the replication rates of the two clones when inoculated on their own. Moreover, Jamonneau *et al*. [31] inoculated *T. b. brucei*/*T. congolense* mixed infection into mice and found that *T. b. brucei* always out competed *T. congolense*, which systematically disappeared.

Reifenberg *et al*. [14] simultaneously infected *G. m. morsitans* with *T. congolense* savannah and *T. congolense* forest and noted the exclusion of *T. congolense* forest from the infection. This might indicate competition, which could occur in the vector gut between the two species simultaneously ingested by the flies. It is acknowledged that future experiments following the approach reported herein should include a number of different strains of *T. b. brucei* and *T. congolense* savannah to ensure that any observed effect is not a strain difference rather than a species related difference.

## Conclusions

The current work shows that qPCR can be used to accurately determine the populations of two different trypanosome species within the tsetse midgut. This will permit researchers to calculate the level of infection of these trypanosomes within the midgut of wild flies where direct microscopic observation is not possible (such as tsetse captured in the wild and stored in acetone before transportation to a laboratory for molecular analysis or those that are homogenised and stored on FTA cards).

The current study also suggests the possibility that a form of competition exists between *T. b. brucei* and *T. congolense* savannah when they co-exist in the fly midgut. However, more research is required to determine the nature of such type of competition between the two species.

## Supporting Information

S1 Fig Average standard curve of cycle threshold (Ct) versus log (10) of *T. b. brucei* concentration/ml; as a single infection (red line where y = -3.5145x + 37.944) and in the presence of *T. congolense* savannah at a concentration of 5 ng per ul (green line where y = -3.3443x + 37.061). Data points were obtained by calculating the mean Ct across sample sets for each standard concentration from 18 reactions, all reactions were spiked with tsetse fly DNA. Vertical bars represent standard deviations
(TIF)

S2 Fig Average standard curve of cycle threshold (Ct) versus log (10) of *T. congolense* savannah concentration/ml; as a single infection (red line where y = -3.5724x + 30.872) and in the presence of *T. b. brucei* at a concentration of 5 ng per ul (green line where y = -3.3428 + 29.354). Data points were obtained by calculating the mean Ct across sample sets for each standard concentration from 20 reactions, all reactions were spiked with tsetse fly DNA. Vertical bars represent standard deviations
(TIF)

S3 Fig Average standard curve of cycle threshold (Ct) versus log (10) of *Glossina* DNA concentration (ng)/μl. Data points were obtained by calculating the mean Ct across sample sets for each standard concentration from 16 reactions. Vertical bars represent standard deviations.
(TIF)

S4 Fig Boxplot for *T. b. brucei* quantification in the four groups (Tb: *T. b. brucei*; sim: simultaneous; Tc: *T. congolense* savannah; seq: sequential). Numbers are presented on the log 10 scale, each boxplot shows the median value and the upper and lower quartiles.
(TIF)

S5 Fig Boxplot for *T. congolense* savannah quantification in the four groups (Tb: *T. b. brucei*; sim: simultaneous; Tc: *T. congolense* Savannah; seq: sequential). Numbers are presented on the log 10 scale, each boxplot shows the median value and the upper and lower quartiles.
(TIF)

## References

[1] Welburn SC, Maudlin I (1991) Rickettsia-like organisms, puparial temperature and susceptibility to trypanosome infection in *Glossina morsitans* . Parasitology. 102: 201–206. 1852487 10.1017/s0031182000062491

[2] Welburn SC, Maudlin I (1992) The nature of the teneral state in *Glossina* and its role in the acquisition of trypanosome infection in tsetse. Ann Trop Med Parasitol 86: 529–536. 1288435 10.1080/00034983.1992.11812703

[3] Mihok S, Olubayo RO, Darji N, Zweygarth E (1993) The influence of host blood on infection rates in *Glossina morsitans* sspp. infected with *Trypanosoma congolense, T. brucei* and *T. simiae* . Parasitology 107: 41–48. 8355996 10.1017/s0031182000079385

[4] Moloo SK, Kutuza SB (1988) Comparative study on the susceptibility of different *Glossina* species to *Trypanosoma brucei brucei* infection. Trop Med Parasitol 39: 211–213. 3194664

[5] Welburn SC, Arnold K, Maudlin I, Gooday GW (1993) Rickettsia-like organisms and chitinase production in relation to transmission of trypanosomes by tsetse flies. Parasitology 107: 141–145. 8414668 10.1017/s003118200006724x

[6] Boulanger N, Brun R, Ehret-Sabatier L, Kunz C, Bulet P (2002) Immunopeptides in the defense reactions of *Glossina morsitans* to bacterial and *Trypanosoma brucei brucei* infections. Insect Biochem Mol Biol 32: 369–75. 11886771 10.1016/s0965-1748(02)00029-2

[7] MacLeod ET, Maudlin I, Darby AC, Welburn SC (2007) Antioxidants promote establishment of trypanosome infections in tsetse. Parasitology 134: 827–831. 17306056 10.1017/S0031182007002247

[8] MacLeod ET, Maudlin I, Welburn SC (2008) Effects of cyclic nucleotides on midgut infections and maturation of *T. b. brucei* in *G. m. morsitans* . Parasit Vectors 14: 5. 10.1186/1756-3305-1-5 PMC2311285 18341697

[9] Peacock L, Ferris V, Bailey M, Gibson W (2007) Dynamics of infection and competition between two strains of *Trypanosoma brucei brucei* in the tsetse fly observed using fluorescent markers. Kinetoplastid Biol Dis 6: 4 17553128 10.1186/1475-9292-6-4 PMC1899512

[10] Gibson W, Ferris V (1992) Sequential infection of tsetse flies with *Trypanosoma congolense* and *Trypanosoma brucei* . Acta Tropica 50: 345–352. 1356306 10.1016/0001-706x(92)90070-e

[11] Kubi C, Van den Abbeele J, Dorny P, Coosemans M, Marcotty T, et al (2005) Ability of trypanosome-infected tsetse flies (Dipetra: Glossinidae) to acquire an infection with a second trypanosome species. J Med Entomol 42: 1035–1038. 16465745 10.1093/jmedent/42.6.1035

[12] Masiga DK, Smyth AJ, Hayes P, Bromidge TJ, Gibson WC (1992) Sensitive detection of trypanosomes in tsetse flies by DNA amplification. Int J Parasitol 22: 909–918. 1459784 10.1016/0020-7519(92)90047-o

[13] Moloo SK, Dar F, Kamunya GW (1982) The transmission of mixed infections of pathogenic *Trypanosoma* species to susceptible hosts by *Glossina morsitans morsitans* . Acta Tropica 39: 303–306. 6131589

[14] Reifenberg JM, Cuisance D, Frezil JL, Cuny G, Duvallet G (1997) Comparison of the susceptibility of different *Glossina* species to simple and mixed infections with *Trypanosoma* (*Nannomonas*) *congolense* savannah and riverine forest types. Med Vet Entomol 11: 246–252. 9330255 10.1111/j.1365-2915.1997.tb00402.x

[15] Mensa-Wilmot K, Hereld D, Englund PT (1990) Genomic organization, chromosomal localization, and developmentally regulated expression of the glycosyl-phosphatidylinositol-specific phospholipase C of *Trypanosoma brucei* . Mol Cell Biol 10: 720–726. 1688997 10.1128/mcb.10.2.720-726.1990 PMC360871

[16] Moser DR, Cook GA, Ochs DE, Bailey CP, McKane MR, et al (1989) Detection of *Trypanosoma congolense* and *Trypanosoma brucei* subspecies by DNA amplification using the polymerase chain reaction. Parasitology 99: 57–66. 2797872 10.1017/s0031182000061023

[17] Maudlin I (1996) Experimental infection of insects: trypanosomes and tsetse. In Crampton, J.M. Beard CB, Kitsos L, editors. The molecular biology of insect disease vectors: a methods manual. pp. 136–145.

[18] Picozzi K, Carrington M, Welburn SC (2008) A multiplex PCR that discriminates between *Trypanosoma brucei brucei* and zoonotic *T. b. rhodesiense* . Exp Parasitol 118: 41–46. 17643434 10.1016/j.exppara.2007.05.014

[19] Welburn SC, Maudlin I (1997) Control of *Trypanosoma brucei brucei* infections in tsetse, *Glossina morsitans* . Med Vet Entomol 11: 286–289. 9330261 10.1111/j.1365-2915.1997.tb00408.x

[20] Becker S, Franco JR, Simarro PP, Stich A, Abel PM, et al (2004) Real-time PCR for detection of *Trypanosoma brucei* in human blood samples. Diagn Microbiol Infect Dis 50: 193–199. 15541605 10.1016/j.diagmicrobio.2004.07.001

[21] Murray W, Peter AT, Teclaw RF (1993) The clinical relevance of assay validation. Compendium on Continuing Education for the Practicing Veterinarian 15, 1665–1676.

[22] Gibson WC, Borst P (1986) Size-fractionation of the small chromosomes of *Trypanozoon* and *Nannomonas* trypanosomes by pulsed field gradient gel electrophoresis. Mol Biochem Parasitol 18: 127–140. 3960051 10.1016/0166-6851(86)90033-2

[23] Van Den Abbeele J, Claes Y, van Bockstaele D, Le Ray D, Coosemans M (1999) *Trypanosoma brucei* spp. development in the tsetse fly: characterization of the post-mesocyclic stages in the foregut and proboscis. Parasitology 118: 469–478. 10363280 10.1017/s0031182099004217

[24] Oberle M, Balmer O, Brun R, Roditi I (2010) Bottlenecks and the maintenance of minor genotypes during the life cycle of *Trypanosoma brucei* . PLoS Pathog 6(7): e1001023 doi: 10.1371/journal.ppat.1001023 20686656 10.1371/journal.ppat.1001023 PMC2912391

[25] Van den Bossche P, De Deken R, Brandt J, Geerts S, Geysen D, et al (2004) The transmission of mixed *Trypanosoma brucei brucei*/*T. congolense* infections by tsetse (*Glossina morsitans morsitans*). Veterinary Parasitology 119: 147–153. 14746974 10.1016/j.vetpar.2003.11.008

[26] Van den Bossche P, De Deken R, Brandt J, Seibou B, Geerts S (2004) Recirculation of *Trypanosoma brucei brucei* in cattle after *T. congolense* challenge by tsetse flies. Vet Parasitol 121: 79–85. 15110405 10.1016/j.vetpar.2004.02.011

[27] Welburn SC, MacLeod E, Figarella K, Duzensko M (2006) Programmed cell death in African trypanosomes. Parasitology 132 Suppl: S7–S18. 17018168 10.1017/S0031182006000825

[28] Welburn SC, Maudlin I, Ellis DS (1989) Rate of trypanosome killing by lectins in midgut of different species and strains of Glossina. Med Vet Entomol. 3(1): 77–82. 2519650 10.1111/j.1365-2915.1989.tb00477.x

[29] Haines LR, Lehane SM, Pearson TW, Lehane MJ (2010) Tsetse EP protein protects the fly midgut from trypanosome establishment. PLoS Pathog. 6(3):e1000793 doi: 10.1371/journal.ppat.1000793 20221444 10.1371/journal.ppat.1000793 PMC2832768

[30] Seed JR (1978) Competition among serologically different clones of *Trypanosoma brucei gambiense in vivo* . J Protozool 25: 526–529. 368326 10.1111/j.1550-7408.1978.tb04179.x

[31] Jamonneau V, Ravel S, Koffi M, Kaba D, Zeze DG, et al (2004) Mixed infections of trypanosomes in tsetse and pigs and their epidemiological significance in a sleeping sickness focus of Cote d'Ivoire. Parasitology 129: 693–702. 15648692 10.1017/s0031182004005876

