# Supplementary figures and images for: Development of Real Time PCR to Study Experimental Mixed Infections of T. congolense Savannah and T. b. brucei in Glossina morsitans morsitans
Source: PLoS One. 2015 Mar 4;10(3):e0117147. doi: 10.1371/journal.pone.0117147 (PMC4349444; doi:10.1371/journal.pone.0117147)

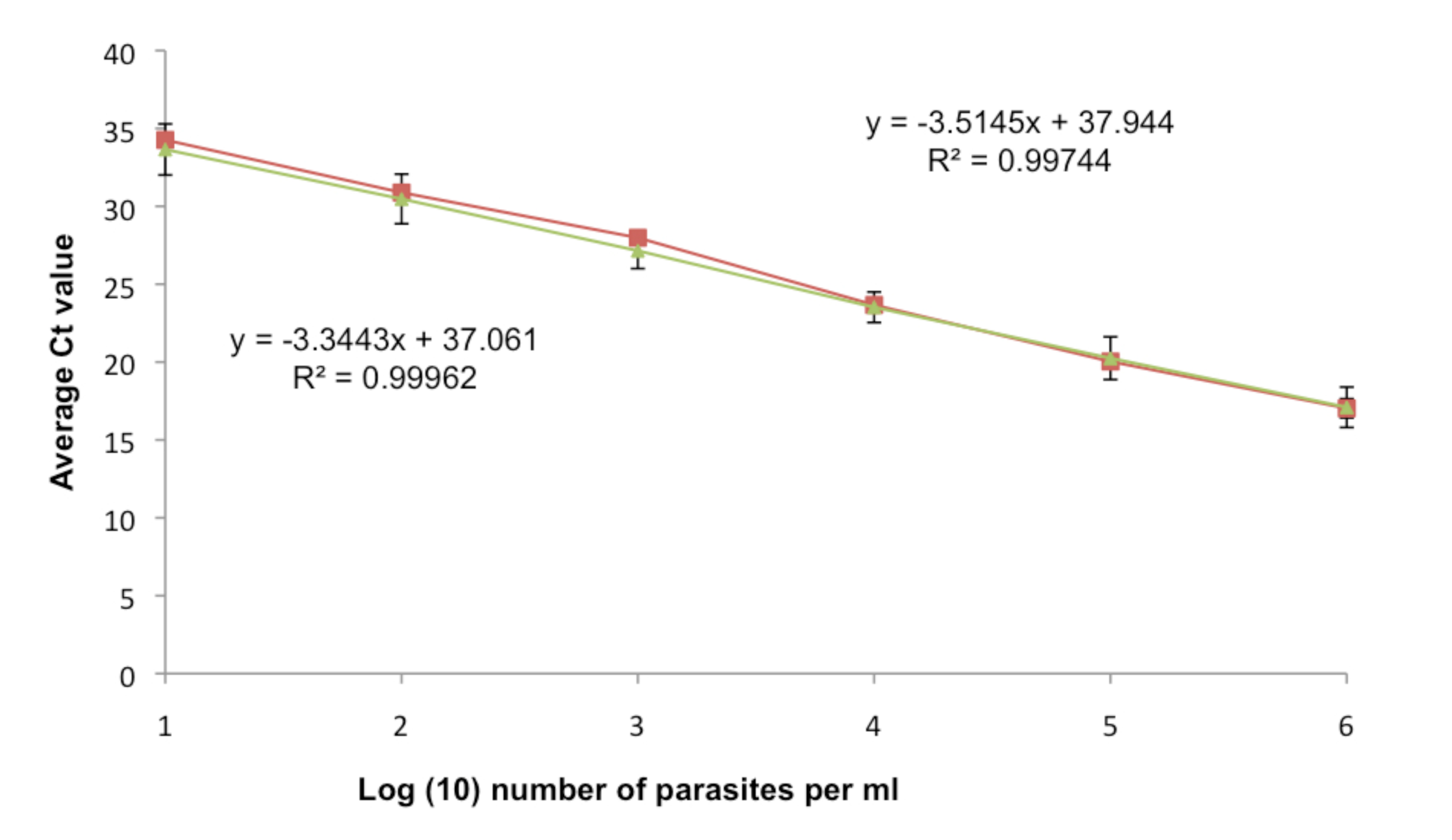

Supplement: S1 Fig — Data points were obtained by calculating the mean Ct across sample sets for each standard concentration from 18 reactions, all reactions were spiked with tsetse fly DNA. Vertical bars represent standard deviations (TIF) [file pone.0117147.s001.tif]

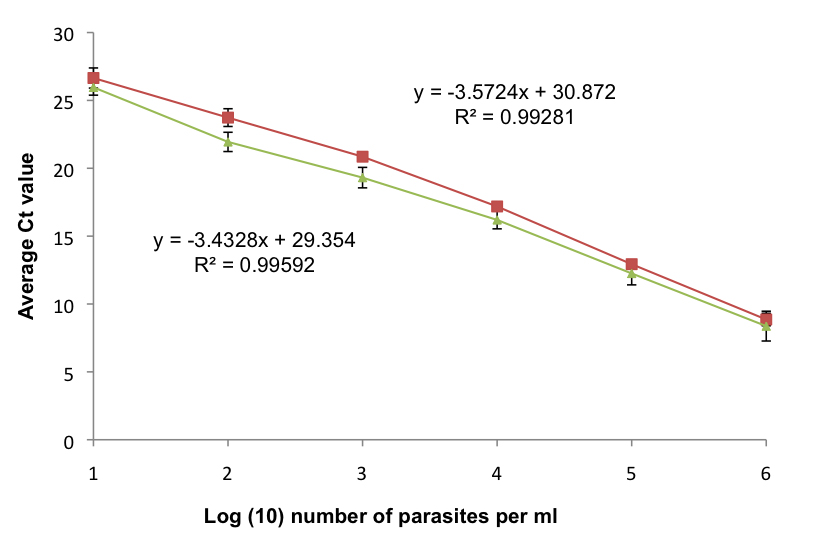

Supplement: S2 Fig — Data points were obtained by calculating the mean Ct across sample sets for each standard concentration from 20 reactions, all reactions were spiked with tsetse fly DNA. Vertical bars represent standard deviations (TIF) [file pone.0117147.s002.tif]

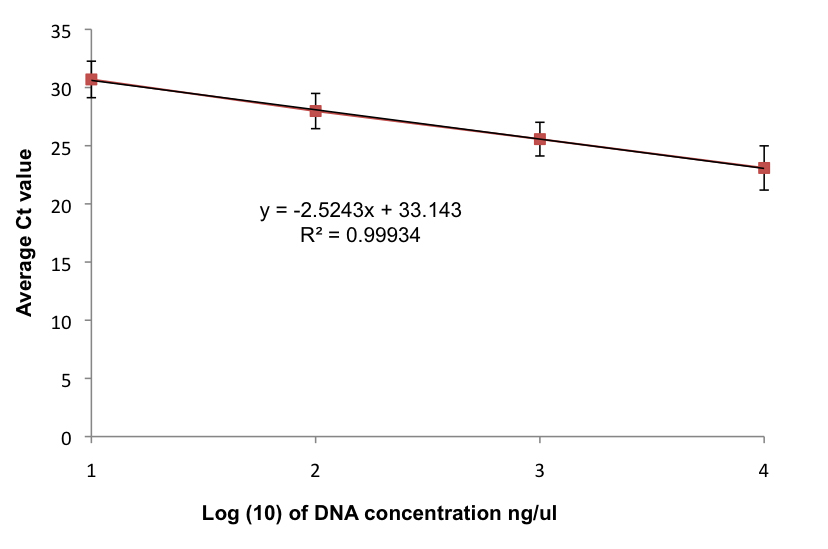

Supplement: S3 Fig — Data points were obtained by calculating the mean Ct across sample sets for each standard concentration from 16 reactions. Vertical bars represent standard deviations. (TIF) [file pone.0117147.s003.tif]

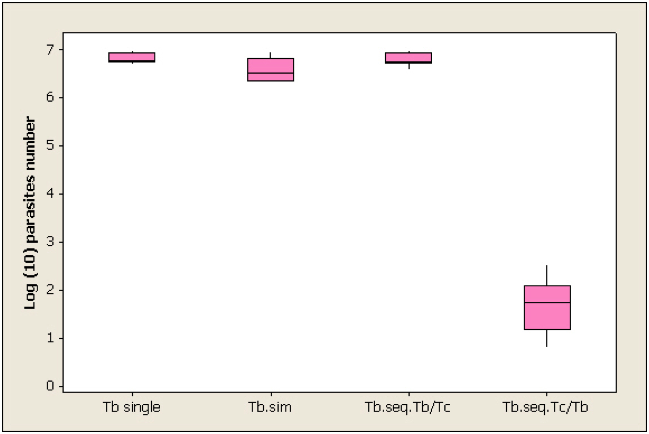

Supplement: S4 Fig — Numbers are presented on the log 10 scale, each boxplot shows the median value and the upper and lower quartiles. (TIF) [file pone.0117147.s004.tif]

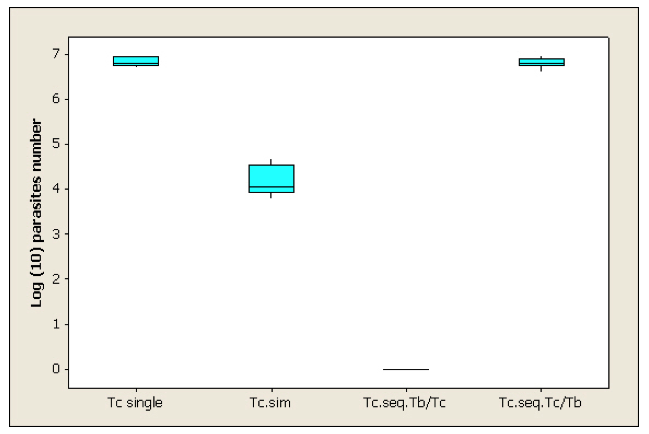

Supplement: S5 Fig — Numbers are presented on the log 10 scale, each boxplot shows the median value and the upper and lower quartiles. (TIF) [file pone.0117147.s005.tif]
